# International cooperation in public health in Martinique: geostrategic utility for cancer surveillance in the Caribbean

**DOI:** 10.1186/s12992-020-00551-w

**Published:** 2020-03-04

**Authors:** Clarisse Joachim, Thierry Almont, Moustapha Drame, Cédric Contaret, Mylène Vestris, Fatiha Najioullah, Aude Aline-Fardin, Patrick Escarmant, Nicolas Leduc, Nathalie Grossat, Xavier Promeyrat, Stefanos Bougas, Eva Papadopoulou, Vincent Vinh-Hung, Emmanuelle Sylvestre, Jacqueline Veronique-Baudin

**Affiliations:** 1 CHU de Martinique, Pôle de Cancérologie Hématologie Urologie, UF 1441 Registre des cancers, Martinique, F-97200 France; 2 CHU de Martinique, Pôle de Cancérologie Hématologie Urologie, UF3636 INTERREG Surveillance du Cancer Oncofertilité, F-97200 Martinique, France; 3 CHU de Martinique, Direction de la Recherche, UF 3603, Unité de Soutien Méthodologique à la Recherche, Martinique, F-97200 France; 4 CHU de Martinique, Direction de la Recherche, UF 3163, Délégation de la Recherche et de l’innovation, Martinique, F-97200 France; 5 CHU de Martinique, Pôle de Biologie, Laboratoire de Virologie, F-97200 Martinique, France; 6 CHU de Martinique, Pôle de Biologie, Laboratoire d’anatomopathologie, F-97200 Martinique, France; 7 CHU de Martinique, Pôle de Cancérologie Hématologie Urologie, F-97200 Martinique, France; 8 CHU de Martinique, Centre de Données Cliniques, F-97200 Martinique, France; 9 CHU de Martinique, Pôle de Médecine, Service de Maladies Infectieuses, F-97200 Martinique, France; 10 INSERM, U1099, F-35000 Rennes, France; 11 Université de Rennes 1, LTSI, F-35000 Rennes, France; 12 CHU de Martinique, Pôle de Cancérologie Hématologie Urologie, UF 3596 Recherche en Cancérologie Hématologie, F-97200 Martinique, France

**Keywords:** Cancer, Cooperation, Epidemiology, Caribbean

## Abstract

**Background:**

Cooperation in public health and in oncology in particular, is currently a major issue for the island of Martinique, given its geopolitical position in the Caribbean region. The region of Martinique shares certain public health problems with other countries of the Caribbean, notably in terms of diagnostic and therapeutic management of patients with cancer. We present here a roadmap of cooperation priorities and activities in cancer surveillance and oncology in Martinique.

**Main body:**

The fight against cancer is a key public health priority that features high on the regional health policy for Martinique. In the face of these specific epidemiological conditions, Martinique needs to engage in medical cooperation in the field of oncology within the Caribbean, to improve skills and knowledge in this field, and to promote the creation of bilateral relations that will help to improve cancer management in an international healthcare environment.

**Conclusions:**

These collaborative exchanges will continue throughout 2020 and will lead to the implementation of mutual research projects across a larger population basin, integrating e-health approaches and epidemiological e-cohorts.

## Introduction

Cooperation is a major component of France’s worldwide public health efforts that aim to improve health for people all over the globe. Far from being limited to humanitarian activities, international hospital cooperation is developing in all hospital-related domains. Because the hospital is at the heart of the healthcare system, international hospital cooperation is one of the means of implementing public assistance targeting health.

Cooperation in public health and in oncology in particular, is currently a major issue for the island of Martinique. Martinique, a region of France, is located in the Lower Antilles of the West-Indies in the Caribbean. The island covers an area of 1128 km^2^ with a population of 383,910 as of 2014 [1]. Given its geopolitical position in the Caribbean region, Martinique shares certain public health problems with other countries of the Caribbean, notably in terms of diagnostic and therapeutic management of patients with cancer [2]. Indeed, there is a high prevalence of chronic diseases (cancer, type 2 diabetes, arterial hypertension, stroke and end-stage kidney failure) in Martinique. As in the neighbouring island of Guadeloupe, Martinique suffers from a pronounced shortfall of women in the 18 to 39 year age bracket, and of men in the 20 to 40 year age group. Accordingly, only 21% of the Martinique population is aged between 20 and 40 years, versus 25% in mainland France. The ageing of the population has accelerated notably in the last two decades in Martinique. In 1990, 68% of the population was aged under 40, compared to only 45% now [3, 4].

In Martinique, a total of 1583 new cases of cancer are recorded per year (all sites considered), of which 61% occur in men, with a male/female sex ratio of 1.5 [5]. With world-standardized incidence rates of 301.6 per 100,000 person-years in men, and 168.4 in women, Martinique counts among the regions of France with the lowest overall incidence of all cancers, along with Guadeloupe and French Guyana. In men, the most common type of cancer is prostate cancer (55%), accounting for more than half of new cancer cases each year, well ahead of colorectal (9%) and stomach (4%) cancer. In women, the most common type of cancer is breast cancer (33%), well ahead of colorectal (14%), stomach and cervical cancer (5% each). A total of 729 cancer-related deaths occurred per year, of which 56% were in men. This situation compares favourably with mainland France (all sites considered) with an incidence that is 15% lower in men and 34% lower in women. Mortality is 18% lower in men and 8% lower in women in Martinique [6].

However, the distribution of these cancers differs from that of mainland France, with large disparities for certain cancer localisations, such as the prostate [7–9], cervical cancer [10], stomach and multiple myeloma.

The ethno-geographic and socio-demographic characteristics in this population of mainly Afro-Caribbean origin could partially explain these disparities. However, other factors also deserve to be investigated, including: 1) increased rates of smoking, particularly in women; 2) Changes in lifestyle and eating habits that have led to a high rate of obesity, a known risk factor for cancer, especially colorectal cancer; 3) Environmental pollution by pesticides, particularly chlordecone, which warrants further research in cancer sites other than the prostate; 4) The role of oncogenic infectious agents also deserves investigation, such as the human papillomavirus and its genotypes circulating in the Caribbean, which are associated with a high risk of cervical cancer; as well as also Helicobacter pylori and the risk of stomach cancer. These factors may be implicated in the pathogenesis or exacerbation of these cancers [11–14].

Successive national cancer plans in France have developed priority actions to be implemented for the management of these patients [15], and these actions can also be integrated into overall healthcare delivery strategies at the level of the Region of Martinique [16, 17], and in the Caribbean in general.

In the face of these specific epidemiological conditions, Martinique needs to engage in medical cooperation in the field of oncology within the Caribbean. Cooperation is necessary to improve skills and knowledge in this field, to pool human and material resources, and to promote the creation of bilateral relations that will help to improve cancer management in an international healthcare environment.

We present a roadmap of cooperative activities in cancer surveillance and oncology in Martinique.

## Main text

### Structuring medical and scientific cooperation in Martinique

#### The main players in international cooperation for Martinique’s development

The international community via the United Nations Organisation has developed 17 Sustainable Development Goals to be achieved by 2030. Health, and its determinants, are mentioned in 12 of these 17 goals. In particular, goal3 (“Good health and well-being”) aims to ensure healthy lives and promote well-being for all, at all ages. France is a member of the Executive Committee of the World Health Organization (WHO), a participating state in the Pan American Health Organisation (PAHO), and an observer in the Regional Office for Africa (AFRO). The partnership agreement signed between France and the WHO defines several priority domains for cooperation, including [1] sanitary security [2]; development goals related to health, and [3] the reduction of risk factors in relation to non-communicable diseases and environmental determinants of health, such as exposure to chemicals, domestic and professional exposure, access to care, education, income, etc. [18].

The countries of the Caribbean are characterized by a number of epidemiological and environmental specificities, as compared with France. These include a higher prevalence of chronic diseases, different population age pyramids, environmental exposure to pollutants, and exposure to extreme meteorological phenomena. France has committed to undertaking cooperative actions in the regions of Martinique, Guadeloupe and French Guyana, that could integrate public health campaigns at the regional level [19, 20]. In the cancer plan, in particular, some actions have been undertaken with the overseas Departments and have already led to the definition of operational objectives for collaborative work in the fight against cancer [15].

At the level of the Caribbean, France has undertaken cooperative programmes with member countries of the Organisation of Eastern Caribbean States (OECS), of which Martinique has been a member since 2015 [21]. The presence of representatives of French research and public health within organisations, platforms and international networks dedicated to health makes it possible to highlight and strategically position France, and French-language expertise in the Caribbean.

Among the major players in medical and scientific cooperation in the field of cancer in Martinique, are the International Cooperation Direction of the University Hospital of Martinique, the Regional Agency for Health, the population based-cancer registry of Martinique (international registry, grade A, created in 1983), the medical divisions of Oncology – Haematology – Urology and the Research Unit for Cancer – Haematology.

To initiate cooperative programmes, it is first necessary to identify medical teams in oncology, and to designate reference medical and scientific persons in charge of cooperation, in the framework of inter-hospital partnership agreements. Cuba, Puerto Rico [22], Dominican Republic as well as the Eastern Caribbean Diagnostic Oncology Network (EC-DON) and the Caribbean Hub of cancer registries are priority targets for cooperative actions. In the long-term, mapping the major players and mapping healthcare pathways for cancer patients in the Caribbean will contribute to widening our knowledge of cancer epidemiology in this zone. These attempts to map healthcare processes in the framework of cooperative efforts in oncology can serve as a model for other medical specialities in Martinique.

Through its presence in large international organisations, both globally and in the Caribbean region, France is striving to create the structural conditions that are amenable to successful cooperation between Martinique and its neighbours.

### Cooperation in cancer surveillance: state of the art in Martinique

#### Healthcare delivery in oncology in Martinique

Within the Caribbean, Martinique counts among the leaders in the development of oncology services thanks to the implementation of a polyvalent and efficient healthcare system that enables all-round, personalized care for patients with cancer.

Hospital admissions in the disciplines of medicine, surgery and obstetrics, expressed relative to the number of inhabitants, are actually lower in Martinique than in metropolitan France. The hospitalization rate varies from 12.4 to 14.1 stays per 100 inhabitants in the French Overseas Departments, compared to an average of 15.7 stays per 100 inhabitants in mainland France. In 2015, the average length of stay in Martinique was slightly higher than in mainland France (respectively 6.2 vs 5.7 days) [23].

The University Hospital of Martinique comprises clinical and medico-technical divisions that cover the main medical, surgical and obstetrical disciplines. The hospital also disposes of beds for dependent elderly persons, and long-term care units. The total capacity of the hospital is 1426 beds, and 120 places, with a total of 5200 employees from a wide range of professions to ensure quality healthcare is delivered. The Oncology – Haematology – Urology division of the University Hospital of Martinique brings together the main healthcare structures for cancer in Martinique, and currently regroups all medical, scientific and paramedical players in the field of oncology. This Division is the reference centre for the treatment of adult cancers in Martinique as regards radiotherapy, medical oncology, haematology and urology. For example, the acquisition of tomotherapy has opened up access to innovative technologies, making it possible to delivery higher doses of radiation to the tumour, while sparing surrounding organs.

#### Martinique and its integration at Caribbean level

The University Hospital of Martinique receives several hundred patients per year from abroad, most of whom come from the countries of the OECS (Dominica, Grenada, Saint Lucia), 54% of them come from Saint Lucia alone [24]. Most are hospitalized for more than 24 h, although outpatient admissions are also increasing steadily. According to diagnosis related groups-based analysis, the most common disease treated in these incoming patients is cancer (outpatient sessions for radiation, chemotherapy, tomotherapy and other techniques). Martinique sees fewer patients leave the island to seek healthcare services elsewhere as compared to Guadeloupe or French Guyana.

In terms of cancer surveillance, the Oncology – Haematology –Urology division of the University Hospital of Martinique received funding in 2018 to create a digital platform for cooperation with existing or future cancer registries in the Caribbean. The objective is to pool data from all Caribbean cancer registries to enhance cancer surveillance. A second phase of this project with clinical implementation of new cancer treatments is emerging.

Through these and other projects, the level of cooperation and integration between Martinique and its Caribbean neighbours is steadily growing.

#### Strategy for cooperation in oncology in Martinique

Cooperative projects have been conducted in the Caribbean through initiatives such as medical cooperation in the Caribbean [25],and networks for cancer surveillance in the region [26, 27]. The theme of cooperation in the field of cancer is a major challenge for the Caribbean countries that require investment for the implementation of cancer care strategies [28]. Multicenter studies integrating data from Caribbean cancer registries have also been conducted, with a view to scientific and international cooperation [29].

In the field of oncology, cooperation involves a wide spectrum of equipment, resources, and reference healthcare options, as well as mobility of physicians, and for many years, has covered the areas of prevention, training and research. This cooperation has been flourishing particularly in border regions that share similar epidemiological characteristics. Historically, from 1981 to 1990, in collaboration with the Ministry for Health, the oncology division and its partners started offering advanced consultations every trimester, and were increasingly called upon to manage and follow-up cancer patients from neighbouring countries. Cooperation between hospitals and universities is one avenue towards increasing the numbers of health professionals, by establishing bilateral partnerships and programmes between countries in the Caribbean. For example, the possibility for medical residents (i.e. specialists in training) from other Caribbean countries to train in Martinique in specialties such as haematology, radiotherapy or public health, makes it possible to maintain a certain level of stability in the healthcare workforce. This is fundamentally important for the fight against cancer, where substantial staff turnover can hamper delivery of care, which may in turn be harmful for patients.

Telemedicine (telepathology and telediagnosis) is currently being widely developed in Martinique. These new possibilities for exchange and communication will enable transverse management of patients at the loco-regional and Caribbean levels for the diagnosis and follow-up of patients with cancer. The problem of medical demographics is pressing, and cooperation between healthcare professionals is a major issue for the Caribbean to enable all countries to benefit from the presence of qualified professionals in neighbouring countries.

Another key issue driving the need for cooperation is to study the epidemiological specificities of the Caribbean region (e.g. high incidence of prostate and stomach cancer and myeloma, exposure to pesticides, effect of obesity…). We need to define shared research focuses in chronic and emerging diseases, and pool our human, financial and material resources through the creation of centres of excellence.

Lastly, childhood cancer care in Latin America and the Caribbean is also a major objective in our region. The PAHO Childhood Cancer Working Group was created in 2017 to support the development of childhood cancer care through structured knowledge exchange, capacity building, and collaboration [30]. The SickKids-Caribbean Initiative was created in 2013 for the care of children with cancer and blood disorders in partnership with the University of the West-Indies, Ministries of Health and health institutions; the aim is to improve outcomes and quality of life for children with cancer [31].

#### Funding for cooperative actions in Martinique

The Ministry for Health, through the Directorate of Health Care Supply (Direction Générale de l’Offre de Soins), finances hospital initiatives through annual calls for projects that attribute funds to healthcare establishments (missions of general interest, and funding for cooperative partnerships). The contribution from each healthcare establishment generally covers the expenses related to welcoming visiting students or healthcare professionals for internships or exchanges.

The territorial authorities, particularly the administrations of individual cities and regions, count among the first-line local partners of healthcare establishments in terms of international cooperation. Hospital-based initiatives taking place in the framework of these partnerships are among the geographical and thematic priorities for decentralised cooperation, and are also important for cohesion policies among local and/or regional authorities. The European Union also offers its own range of financing possibilities, as well as specific geographic instruments such as the European Development Fund, or programmes for cross-border and inter-regional cooperation (INTERREG). The INTERREG programme is funded by the European Regional Development Fund, and aims to promote cooperation between European regions to achieve greater cohesion between member states in terms of public policy and private initiative [32]. There are three types of programmes, namely cross-border cooperation programmes, transnational cooperation programmes, and interregional programmes.

The University Hospital of Martinique is active at the international level in conjunction with the University of the French West-Indies. This partnership takes several forms, including for example shared procedures, exchange of practices and information, joint reception of foreign delegations, implementation of joint missions or projects, and bilateral communication regarding inward and outward mobility of medical personnel holding academic posts.

The main issues at stake in terms of cooperation in cancer for the University Hospital of Martinique are the continuing medical education of its healthcare professionals, sharing of experience, access to innovative therapies and new technologies, as well as the development of networks of competence.

#### Global issues in international cooperation and obstacles to cooperation

##### Human relations

The majority of the difficulties with cooperation arise from the fact that human relations can be complex, socio-cultural environments differ, and healthcare and demographic contexts are heterogeneous across the Caribbean. These differences can complement each other, but also raise issues that need to be taken into account to optimise cooperative missions. The impact of a project on the key stakeholders must be evaluated in advance, as well as after completion of the cooperation.

The availability of healthcare staff is a major issue. Indeed, structuring a network for cooperation relies on the healthcare staff working within the University Hospital of Martinique, which in turn implies that these staff members be released from their ordinary duties to perform missions outside of Martinique in the framework of the cooperation. The healthcare teams need to have enough staff members to make it possible to attend to the needs of the other partners and contribute to the success of the project.

Considering that the Caribbean area is largely English-speaking, Martinique must make a concerted effort to find its place among partners that do not speak French. The healthcare professionals involved must therefore be proficient in comprehension of oral and written English to enable clinical examinations or exchanges with administrative or diplomatic personnel, but also to speak publicly in international scientific meetings (press conferences, congresses, scientific articles). The language barrier is a major obstacle that can be removed by providing training to healthcare professionals in cooperative missions on site in partner countries. Furthermore, cooperative efforts cannot be designed as “person-dependent” initiatives, given the high turnover of medical personnel in the French West-Indies, and the concept of dedicated teams is important for a long-term perspective.

##### Organisational barriers

Our experience has brought us into contact with a range of different types of cooperation projects, and organisational constraints are common to all missions. Indeed, every mission requires at least 6 months of preparatory groundwork to deal with regulatory demands (visas, ministerial authorisations), and ensure availability of the teams, but also to organise an agenda for the partnership work that will maximise the utility of the mission for all the stakeholders involved. Nonetheless, experience has shown that even missions organised in emergency contexts, by an experienced team with the necessary qualifications to manage emergencies and diplomacy, can be sufficiently satisfactory. In emergency situations, the teams are under pressure to optimize management of the resources allocated, and meet objectives that must be clear and achievable.

##### Financial barriers

Managing the financial constraints is an essential part of the preparatory work for any cooperative mission, given the absence of dedicated funding for cooperation in the course of routine hospital activity in France. Apart from funding opportunities such as grants for missions of general interest, or projects funded through the INTERREG programme, few teams avail of funds for cooperation. The role of associations working to promote scientific research can be important in multicentre projects. Non-governmental organisations can also provide support for major events.

#### Risk management: taking account of a force majeure

There are a number of unexpected events that may profoundly affect project management. In around 60% of cases, the likelihood of such events can be studied in advance. In this regard, the Caribbean is at risk of extreme meteorological events including cyclones, earthquakes and to a lesser extent, tsunamis. The risk is at its greatest during the rainy season, i.e. from the end of June to October. During these months, teams should avoid planning any large-scale cooperative missions, given the prohibitive cost of cancellations. Similarly, plane accidents can perturb air traffic and/or lead to re-routing of flights back to France or towards the United States, thereby increasing the costs and duration of transit inestimably.

Hurricane Maria (a Category 4 storm in 2017) is a prime example illustrating the vulnerability of the Caribbean area to high-impact weather-related events, after which cooperation in terms of humanitarian aid becomes the priority for healthcare establishments.

Other unpredictable risks include anecdotal events such as losing one’s passport prior to an international flight, an acute health event or accident on site requiring repatriation in a wheelchair, or detention at customs at entry into a country because of incomplete administrative procedures. The lessons learned from these situations make it possible to improve and better plan for future missions. Above all, these experiences underline that a key player in resolving cross-border incidents is the French embassy (for issuance of an emergency passport, for aid with repatriation etc.). Therefore, it is fundamentally important to inform the French embassy at the destination about the mission and its participants, to anticipate any unforeseen incidents that may require diplomatic intervention.

### Perspectives for development and integration of innovative cooperative projects

#### Development of e-health and innovation as tools for cooperation

The healthcare sector is a major source of attractivity and influence. Excellence in healthcare training for public health greatly boosts attractiveness and opportunities for collaboration in an environment where pooling of resources (human and material) is strongly encouraged. E-health is defined as the application of information and communication technologies to the field of health and well-being, while m-health (i.e. via smartphones and other connected devices) is currently offering innovative opportunities for the development of collaborative projects with patients, patient associations and healthcare professionals in interaction with healthcare provision services.

The growing bilateral relations between OECS countries will be an important lever in enhancing the attractiveness of the region for young researchers and healthcare professionals, through the creation of a centre of attraction, namely a digital platform for medical research and exchange, under the auspices of the Martinique General Cancer Registry and the Oncology – Haematology – Urology division of the University Hospital of Martinique.

This innovative, collaborative data platform, the “Martinique Cancer Data Hub”, comprising both e-health and e-learning options, will provide dedicated interconnected interfaces and an integrated database system, offering a unique experience and outstanding learning opportunities for healthcare professionals, patients and their relatives as well as regional health authorities. Each participant will contribute to a particular set of values, with an overall architecture that guarantees the quality and security of integrated health data management.

In the long term, this should facilitate surveillance and data collection at a population level, yet using a person-centred approach. In view of the private nature of the data concerned, an essential preliminary step is to lay down a strict legal framework for technological innovations in health, before moving forward with large-scale projects in multicentre initiatives with other countries of the OECS or Caribbean.

The objective of the interactive “Martinique Cancer Data Hub” platform is to enable efficient analysis of health data for surveillance and research in oncology and haematology. This secure internet platform plans to:

- Create e-cohorts of patients for the main cancer types (prostate, breast, colorectal…);
- Model the life pathways and aging pathways of patients according to treatment type, age at diagnosis or cancer stage;
- Investigate overall and specific quality of life within these cohorts;
- Propose projects in the field of pharmaco-epidemiology, to evaluate the real-life efficacy of treatments;
- Create a consortium of researchers (mapping the major players in the field), and disseminate their scientific publications;
- Inform and recruit patients who might participate in innovative clinical trials, putting the patient at the heart of the research.

Through these innovative initiatives, existing working partnerships between our medical and scientific teams and our international colleagues will be strengthened and cemented. This project will provide answers to priority issues in the field of health, and will also promote French expertise in this area.

#### Quality of life and cancer: example of a highly relevant topic for scientific cooperation in the field of cancer

Incidence and mortality from prostate cancer in Martinique [7, 33] count among the highest in the world. With this in mind, we developed a collaborative project focused on overall quality of life, and quality of sexual health [8], by implementing quality of life questionnaires in routine practice during the constitution of a cohort of patients with prostate cancer. Health-related quality of life is a multidimensional concept that encompasses the physical, mental and social domains, but also biological and clinical domains. To implement federative projects in the field of cancer in the elderly in the framework of healthcare cooperation across the Caribbean, a Task Force was created to strengthen cooperative efforts between cancer registries [22]. This consortium of researchers, all specialized in cancer surveillance, plans to implement larger scale multicentre epidemiological studies, as well as clinical trials and quality of life studies in cancer patients.

**Fig. 1:**
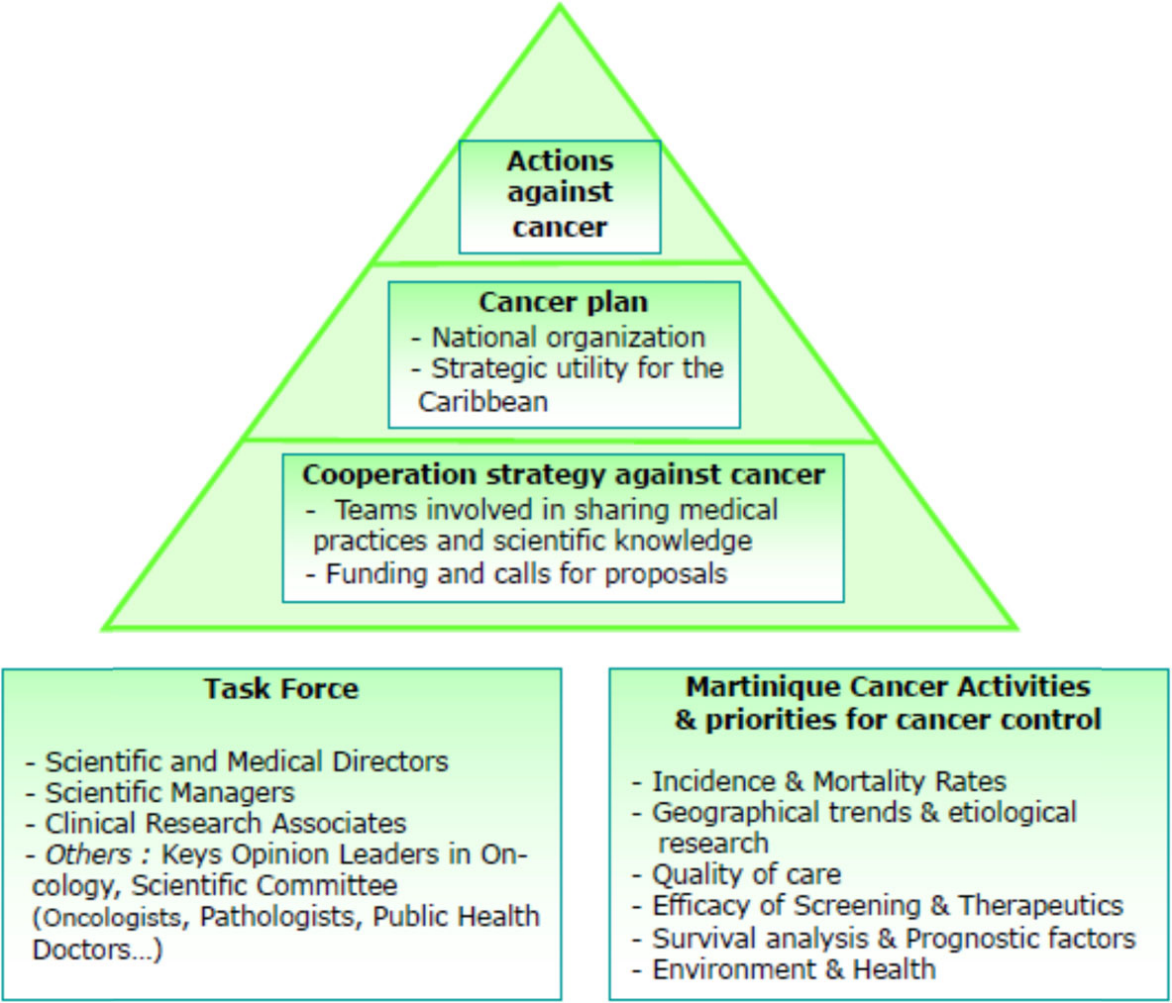
Scientific cooperation in the field of cancer in Martinique

In this regard, the Cancer Registry of Martinique has proposed a roadmap for the set-up of a network for scientific, technical and clinical collaboration in cancer in the Caribbean. Other areas such as communication, methodological support and teaching could also be addressed within the network. In this way, data from France’s territories in the Caribbean could be utilized for scientific purposes, while also creating a bridge between the Caribbean and the Franco-European zone (Fig. 1).

#### Utility of integrated action in a strategic plan for cancer surveillance in the Caribbean

The International Agency for Research on Cancer (IARC) has established Regional Hubs to oversee activities with the leading countries in their catchment area. The aim is to help the countries in the Hub to develop their own plans to fight cancer, by providing methodological support and by helping them to acquire the necessary knowledge. In each region, the core activities comprise:1) Providing local training courses and individualized support; 2) Promoting research; 3) Assisting in the development of regional networks and facilitating communication. According to information available on the Global Initiative for Cancer Registry Development (GICR) [34], the GICR will be operational in 20 low and middle-income countries by 2020, and 30 additional countries by 2025. At a regional level, a support centre is on-going for all the cancer registries of the Caribbean with the creation of the Caribbean Hub, and the contribution of the Martinique Cancer Registry has been highlighted through a partnership agreement with the GICR, in view of the high quality of this registry within the Caribbean.

Scientific collaboration for cancer surveillance in the Caribbean through a regional network of cancer registries was initiated with the advent of the registry in 1983. On-site visits started in the 1990s, and the most recent visits in 2015, 2016 and 2017have helped renew cooperation with Puerto Rico and Cuba, and promote partnerships with other countries of the OECS, as well as with the cancer registries of Guadeloupe and French Guyana. A roadmap has been developed, with step-by-step plans to achieve an optimal cancer research system at local level (in Martinique), at a regional level (across the French West-Indies) and at national and international levels.

Since the Region of Martinique joined the OECS, the example of the cancer registry network as a model of cooperation for cancer within the Caribbean has been presented at the first conference of Ministers for Health of the OECS member countries in 2014 in Saint Vincent. Martinique’s geographical position enables it to propose support for cancer registry development in the neighbouring islands. Thanks to this project, it is hoped that improvements in epidemiological knowledge and public health planning will be achieved. Cancer registries currently provide high-quality data about incidence and mortality at international level (within the IARC). Cooperation with the OECS countries and the wider Caribbean area would meet the desired objectives in terms of support for the development of cancer surveillance activities, and training of healthcare professionals [35] through the creation of a hub for the development of cancer registries [36].

## Conclusion

Collaborative projects aimed at sustaining cancer research activities in the Caribbean, through the development of a digital platform, will help to identify the clinical, demographic, socio-economic and organisational determinants that give rise to the heterogeneity in cancer across the Caribbean. Thanks to the cooperative missions already carried out, the General Cancer Registry of Martinique has strengthened cooperation with Puerto Rico, Cuba, Dominican Republic, Saint Lucia, as well as with Guadeloupe and French Guyana in the context of the French Network of Cancer Registries. The full spectrum of these cooperative initiatives carried out between 2015 and 2019 have been presented in international scientific meetings, and have led to renewed cooperation with the registries of the Pan-American zone in the framework of working groups. These collaborative exchanges will continue throughout 2020 and will lead to the implementation of mutual research projects across a larger population basin, integrating e-health approaches and epidemiological e-cohorts.

## Data Availability

Not applicable.

## Abbreviations

AFRO: Regional Office for Africa of the World Health Organization
GICR: Global Initiative for Cancer Registry Development
IARC: International Agency for Research on Cancer
INTERREG: European Union funding programme for cross-border and inter-regional cooperation
OECS: Organisation of Eastern Caribbean States
PAHO: Pan American Health Organization
WHO: World Health Organization
